# A Case of Multiple Endocrine Neoplasia Type 2B and Gangliomatosis of Gastrointestinal Tract

**DOI:** 10.1155/2012/491054

**Published:** 2012-10-08

**Authors:** Banafshe Shahnazari, Aria Aghamaleki, Bagher Larijani, Mohammad Reza Mohajeri Tehrani, Hasan Rafati, Abdolreza Babamahmoodi

**Affiliations:** ^1^Endocrinology and Metabolism Research Institute, Tehran University of Medical Sciences, Tehran, Iran; ^2^Health Management Research Center, Baqiyatallah University of Medical Sciences, Tehran, Iran

## Abstract

Multiple endocrine neoplasia type 2 (MEN2) is a rare familial syndrome caused by mutations in the RET protooncogene and it is transmitted as an autosomal dominant trait. The underlying problem for all the MEN syndromes is failure of a tumour suppressor gene. The genetic defect in MEN2 is on chromosome 10 (10q11.2) and has also been identified both for MEN2A and MEN2B. The reported patient is an 18-year-old girl presented with long-term diarrhea and enterocutaneous fistula. Her thyroid nodules, marfanoid habitus and bumpy lips, were also highly suggestive for MEN2B.

## 1. Introduction

Multiple endocrine neoplasia type 2 (MEN2) is a rare familial syndrome caused by mutations in the RET protooncogene. The underlying problem for all the MEN syndromes is failure of a tumour suppressor gene. The genetic defect in MEN2 is on chromosome 10 (10q11.2) [1]. MEN type 2 is transmitted as an autosomal dominant trait associated with various endocrine tumors. It has been subcategorized into two major syndromes called MEN2A and MEN2B [2, 3].

Each of the two major types of multiple endocrine neoplasia affects an estimated 1 in 30,000 people [1]. Among the subtypes of multiple endocrine neoplasia type 2, type 2A is the most common form, followed by familial medullary thyroid cancer. Type 2B is relatively uncommon, accounting for about 5 percent of all cases of multiple endocrine neoplasia and reported in approximately 1000 families worldwide in 2001. Men 2B is much less common but also more aggressive and is associated with medullary thyroid cancer (MTC), pheochromocytoma, multiple mucosal neuromas, gangliomatosis of gastrointestinal tract, and a marfanoid habitus, whereas hyperparathyroidism is absent [2–7].

Patients with MEN2B can present with various manifestations: one might be diagnosed due to mucosal neuromas in childhood; one might present with symptoms related to phechromocytoma or ganglioneuromatosis of the GI tract which may cause diarrhea, intestinal obstruction, and colicky pains.

The patient described here had long-term diarrhea and presented with enterocutaneous fistula. Her thyroid nodules, marfanoid habitus and her bumpy lips, were noticed by one of our colleague as highly suggestive for MEN2B. 

In contrary to the case being reported here who presented with diarrhea, patients with MEN2B usually have other presentations.

## 2. Case Report

An 18-year-old girl presented with long-term diarrhea and enterocutaneous fistula. She was diagnosed with celiac disease 4 years ago, because of nonbloody low volume, yellow colored diarrhea with tenesmus occurring 3-4 times a day. She was given the appropriate diet for celiac disease.

Three months before admission, she developed pain and swelling in the left lower quadrant of the abdomen and erythema of the same area. 

She underwent surgery with probable diagnosis of abscess or inguinal hernia, but she had no clinical documents of the results of the surgery. 45 days later, a fistula was formed at the site of surgery which had fecaloid secretions. 

She also had primary amenorrhea and a history of hypothyroidism. 

On examination, she had a marfanoid habitus, a tall stature, and increased joint laxity. Blood pressure was 90/60 mmHg. 

**Figure 1:**
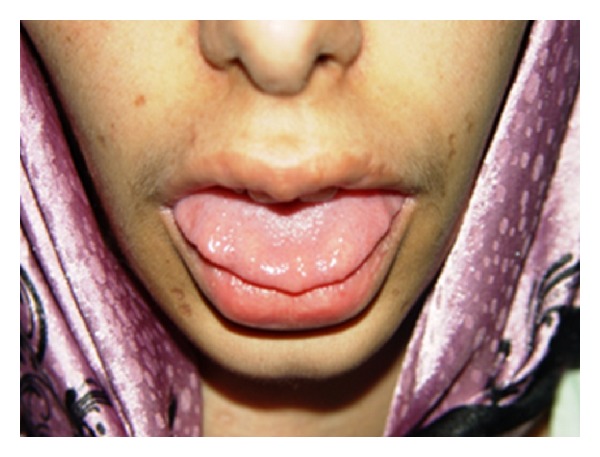
The patient had mucosal neuromas on the anterior third of her tongue. Note the bumpy/blubbery lips.

Bumpy lip/neuromas were found on the distal part of the tongue as well as on the conjunctiva of both eyes (Figure 1). 

**Figure 2:**
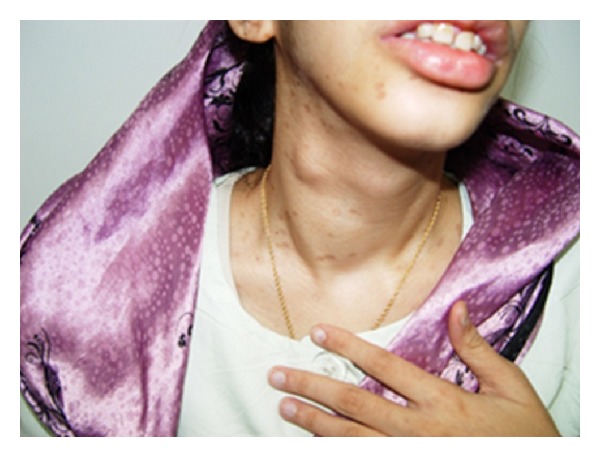
On physical examination, she had goiter with large nodule in the right lobe of her thyroid gland.

Thyroid examination revealed multinodular goiter with a large nodule (4 ∗ 1.5 cm in diameter) in the right lobe of the thyroid gland with lymphadenopathy (Figure 2). Physical examination was otherwise normal.

Laboratory data was as follows:

 24 hrs urine metanephrine: 192 mcg/24 hr (normal: <350),
 24 hrs urine normetanephrine: 93 mcg/24 hr (normal: <600),
 Hb: 13,1, WBC: 16600, Plt: 533 ∗10^3^,
 ESR: 22,
 serum albumin: 4,4 g/dL (3,5–5),
 cholesterol: 145 mg/dL, HDL: 51 mg/dL, TG: 175 mg/dL,
 iron: 71, TIBC: 406,
 24 hrs urine calcium: 66 *μ*g/dL,
 antiendomysial antibody: 0.1,
 BUN: 7, Cr: 0,7,
 Anti-TPO: 14,3,
 LFT: NL,
 antitTG IgA: 1,4 (−),
 calcitonin: >2000 (with normal limit up to 140),
 CEA: 3689 (with normal limit up to 37).

In consultation with endocrinologists, MEN2B was suggested due to the thyroid nodule, cervical lymphadenopathy, marfanoid appearance, mucosal neuromas, and gastrointestinal tract abnormalities.

**Figure 3:**
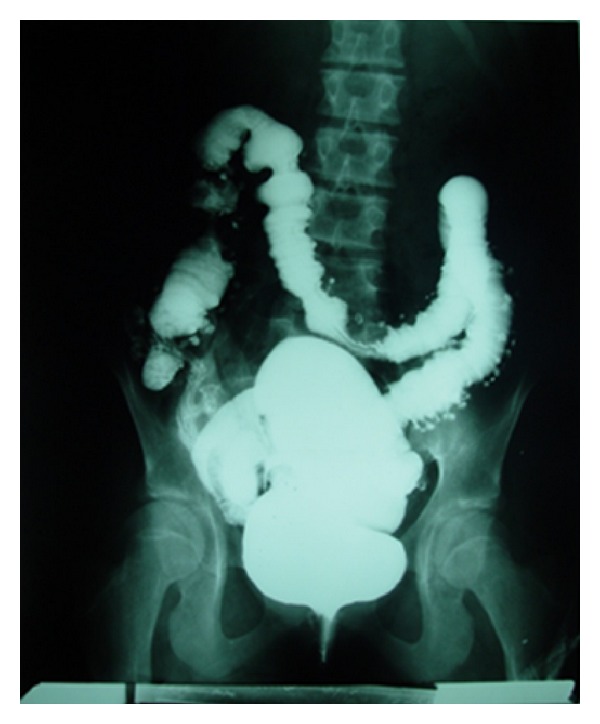
Barium enema of the patient revealed diverticulosis of the sigmoid colon and left colon.

Barium Enema revealed diverticulosis of sigmoid colon (Figure 3). 

Colonoscopy revealed hypervascular areas with several fistulae orifices in the sigmoid colon.

Thyroid ultrasound revealed multinodular goiter, one large nodule, and cervical lymphadenopathy.

EUS revealed several calcified lesions in the liver and multiple round hyperechoic lesions in the liver suggesting metastasis of MTC.

**Figure 4:**
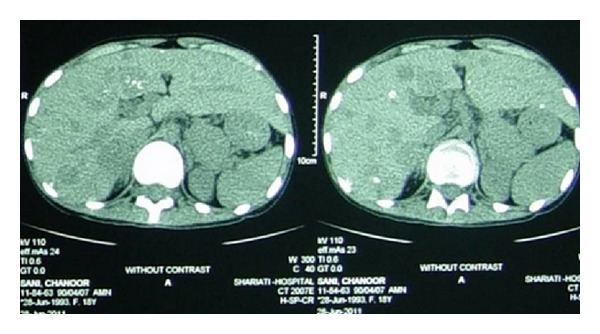
Spiral abdominopelvic CT scan (with contrast). The report was as follows. Multiple calcified and noncalcified lesions in liver are seen (metastasis should be considered). Some of the small bowel loops have thickened wall. Mild right side hydronephrosis is present. Anterior abdominal wall fistula is depicted. A few small paraaortic lymphnodes are seen. Mild left side pleural effusion and massive ascites were also noted.

Abdominal CT scan revealed abscess formation in the site of previous surgery. Multiple calcified and noncalcified lesions were seen in the liver, suggestive of metastasis (Figure 4). 

Small bowel transition was normal.

Ophthalmologic consultant reported sclerotic neuropathy and large corneal nerves.

Fine needle aspiration of the thyroid nodule reported medullary thyroid carcinoma. Both lobes were involved; capsular invasion and lymphatic vessel invasion were noted. IHC staining was performed on the blocks; the results were as follows:

1. TTF1: negative in tumor cells,
2. calcitonin: positive in tumor cells,
3. chromogranin: positive in tumor cells,
4. synaptophysin: positive in tumor cells.

Genetic investigation: exon 16/RET mutation at codon 918. The patient has the following nucleotide changes:

1. heterozygote T2307G in exon 13 which causes no AA change (Leu769leu),
2. heterozygote T2753A in exon 16 which causes Met918Thr.

Change number 1 is a polymorphism in RET gene. Mutation in exon 16 is a common mutation that is found in 95% of MEN2B patients.

She went under total thyroidectomy and excision of the left anterior cervical lymphatic chain.

One week later, she was admitted again for the right lymphatic chain excision. She still had fecaloid secretions. One week after that, she was operated for the fistula and sigmoidectomy and appendectomy were performed.

## 3. Discussion

Hereby, we reported a case in whom long-term diarrhea was the main clinical manifestation that occurred in setting of MEN2B. Intestinal involvement in MEN2B is characterized by ganglioneuromas that can affect anywhere in the gastrointestinal tract [4]. This pathology causes loss of normal bowel tone, diverticulosis, colonic distension, and mega colon [8, 9]. Sometimes, intestinal involvement is so severe that surgical interventions such as partial colectomy are required [10].

Our patient was diagnosed with MEN2B in the age of 18; however, patients with MEN2B usually present in the first decade of life [6]. Recent studies revealed that the age at diagnosis could range from 1 to 31 years old [7]. Marfanoid appearance—also present in our patient—is found in 75% to 100% of patients with the disease [11]. Other skeletal abnormalities such as kyphosis, pectus excavatum, and talipes supinatus are also seen. Blubbery and bumpy lips are other typical characteristics of MEN2B individual as seen in our patient. Mucosal neuromas reported in 100% of patients were also noted [12]. They are often described as painless sessile nodules on the lips or tongue. Similar nodules are also seen in the sclera and eye lids. 

MTC is almost always reported in patients with MEN2B, though it is a very rare type of thyroid cancer [13]. Our patient had the most common presentation of MTC: thyroid nodule. It is either single or multiple. The prevalence varies from 5 to 10% among thyroid cancer and 0.4 to 1.4% among thyroid nodules [14, 15]. This cancer usually appears later in sporadic cases [2]. High calcitonin levels in urine and serum are due to MTC [16]. 

MTC has a poorer prognosis in patients with MEN2B, when compared to the sporadic cases or in those with MEN2A [17]. Distant metastasis also exacerbates the prognosis which was noted as metastatic lesions in the liver in our patient. Only advanced metastasis can cause diarrhea and flushing in patients [18]. Chemotherapy or radiation is not affective in these patients. Due to the progressive pattern of MTC in these patients, prophylactic total thyroidectomy is highly recommended when the RET mutation is detected [18]. According to the fact that thyroid cancer may even metastasize in the first decade of life, the ideal age proposed for surgery is four years and even less (one year) [10, 19]. 

Pheochromocytoma is another manifestation of the disease which affects 50% of the patients. Our patient did not have this manifestation as a part of her disease. 

The clinical diagnosis of MEN2B was confirmed in our patient by the genetic analysis showing the typical RET mutation at codon 918. This specific mutation is found in over 95% of patients with MEN2B [4, 10, 17]. 

They may occur by dual (tandem) mutations 804 and 806 or 804 and 904 [20]_._ A RET protooncogene mutation A883F displays a more indolent form [21–23].

However, the gene responsible for MEN type 2 was discovered many years ago and some advances in better treating multiple endocrine neoplasia type 2-associated conditions have occurred over the last decade (for instance, tyrosine kinase inhibitors); the exact mechanisms of tumor development in patients affected with RET germ line mutations remain unknown. Recent researches suggest that an overrepresentation of mutant RET as a “second hit” event might trigger tumor genesis. However, alterations in other genes might contribute to this overrepresentation of RET or impact on MEN 2-related tumor development through completely different mechanisms and pathways. In one study there is a suggestion about duplication of the mutant RET allele in trisomy 10 or loss of the wild-type allele in multiple endocrine neoplasia type 2-associated pheochromocytomas.

The final goal of further elucidating the natural history and pathogenesis of MEN2-related tumors should be the chance to offer patients with RET germ line mutations an optimal cancer prevention and treatment program [24–26].

In conclusion, hereby, we reported a case of MEN2B with long-term diarrhea as the main concern of the patient. The patient was not even admitted in the endocrinology ward. However, gastrointestinal symptoms, associated with marfanoid habitus, mucosal neuromas, and thyroid nodules which proved to be MTC after further investigation were highly in favor of diagnosis with MEN2B. Genetic analysis confirmed the diagnosis. Based on our experience, more accurate attention to the general appearance of the patients and a precise physical examination could reveal the settings in which chronic manifestations take place, and the clinician might be able to prevent the irreversible aftermaths of the disease and save the patient from disastrous complications.
